# Continuous Measurement of Three-Dimensional Root Canal Curvature Using Cone-Beam Computed and Micro-Computed Tomography: A Comparative Study

**DOI:** 10.3390/dj8010016

**Published:** 2020-02-06

**Authors:** Michael Kucher, Martin Dannemann, Niels Modler, Dominik Haim, Christian Hannig, Marie-Theres Weber

**Affiliations:** 1 Institute of Lightweight Engineering and Polymer Technology (ILK), Technische Universität Dresden, Holbeinstraße 3, 01307 Dresden, Germany; michael.kucher@tu-dresden.de (M.K.); niels.modler@tu-dresden.de (N.M.); 2 Clinic of Oral and Maxillofacial Surgery, Medical Faculty Carl Gustav Carus, Technische Universität Dresden, Fetscherstraße 74, 01307 Dresden, Germany; dominik.haim@uniklinikum-dresden.de; 3 Clinic of Operative and Pediatric Dentistry, Medical Faculty Carl Gustav Carus, Technische Universität Dresden, Fetscherstraße 74, 01307 Dresden, Germany; christian.hannig@uniklinikum-dresden.de (C.H.); marie-theres.weber@uniklinikum-dresden.de (M.-T.W.)

**Keywords:** Three-dimensional curvature, molar, distal root canal, cone-beam computed tomography (CBCT), micro-computed tomography (µCTmicro-CT), geometry, morphology, volume model

## Abstract

The knowledge of root canal curvature is crucial regarding the prevention of ledge formation, root perforation and the possibility of endodontic instruments’ fracture during endodontic treatments. Therefore, a quantification method of the root canal curvature as well as the applicability of diagnostically relevant tomographic three-dimensional (3D) imaging data is necessary. Hereby, cone-beam computed tomography (CBCT) images and micro-computed tomography (µCT) data of distal root canals were analysed concerning the continuous three-dimensional curvature of human mandibular molars (n = 50). The curvature of the canal’s three-dimensional centre line was determined by evaluating the tomographic images. The centroids of each root canal slice were identified and approximated by spline curves to obtain the centre line and therefore, its curvature. Comparing the results evaluated from CBCT and µCT images, minimum radii of curvature of 2.6 mm and 2.1 mm were determined, respectively. The observation of the centre line demonstrated the requirement of the three-dimensional imaging data from CBCT and µCT for a reliable curvature analysis. Conclusively, the evaluation of CBCT and µCT images results in comparable radii of curvature. Thus, the application of the introduced method in combination with CBCT applied to patient cases could offer an important preliminary diagnostical step to prevent endodontic treatment complications.

## 1. Introduction

Extensive knowledge regarding root canal anatomy, especially its curvature, is essential for the successful endodontic treatment of teeth or even the design of endodontic instruments. A quantification of the root canal curvature is essential to assess the risk of ledge formation [1], root perforation [2] or instrument fracture of rotary files as well as irrigation tips during root canal treatment [3]. The quantification enables the determination of instrument stress that affects the instruments’ fatigue life. Furthermore, the knowledge of the canal curvature helps to assess the difficult root canal sections leading to a better root canal preparation as well as bacterial decontamination and prevents possible perforation or step formations that can severely decrease the treatment success.

Firstly, Schneider investigated the curvature of human root canals by evaluating the angle between two lines on roentgenograms which represented the first one-step curvature measurement [4]. Further studies measured the curvature by means of one or more angles between different defined lines (see References [5,6,7]). Another approach was the determination of the radius of curvature, as a circular approximation of the root canal’s centre line from two- as well as three-dimensional images (compare Reference [7]). Peters et al. [8], Lee et al. [9,10], Park et al. [11] and Dannemann et al. [12] measured the curvature from three-dimensional (3D) centre lines and obtained the continuous root canals curvature along the canal length. This promising approach enabled a reliable quantification of curvature for root canals which were significantly curved in the buccolingual as well as mesiodistal direction running from the apical foramen to the floor of the pulp chamber.

The determination of the centre line can be obtained by morphological thinning [13] or by the connection of root canal cross-sections’ centres. The centres were calculated from the volume model’s slices perpendicular to the longitudinal axis of the tooth. Most of the studies used the centre of gravity [8,12,14,15,16]. Other approaches were the usage of the intersection point of the root canal’s major and minor axis [9,10,11], the manual detection of centre points with the three-dimensional image data [17] or their evaluation of mesiodistal and buccolingual two-dimensional (2D) radiological views [18]. However, to obtain correct measurements, a three-dimensional centre line is required [8,9,10,11,12,13,14,15,17,19]. To minimise fluctuation and incorrect detection of centre points, the centre lines were approximated by three-degree polynomial functions [20], fourth-degree polynomial functions [18,19] or spline curves [9,10,14]. Dannemann et al. figured out that the minimal radius of curvature depended on the polynomial degree of the approximation [12].

Three-dimensional tomographic imaging techniques are required to obtain a three-dimensional curvature measurement. Imaging by means of micro-computed tomography (µCT) systems result in high-resolution topographical images and obtain volume models with high level of details. Nevertheless, µCT imaging can only be used to study extracted teeth [21]. Cone-beam computed tomography (CBCT) enables a visualisation of root canal morphology in vivo [22] and is already established for endodontic treatments.

The aim of this study was (1) to introduce a method for a reliable continuous three-dimensional measurement of root canal curvature along the longitudinal root canal axis and (2) to demonstrate the applicability of CBCT systems to evaluate root canal curvatures. Therefore, a comparative study evaluating the three-dimensional imaging data from µCT and CBCT by means of the introduced curvature measurement method was performed and the resulting curvatures of distal root canals (n = 50) from human mandibular first and second molars were evaluated and compared. For this comparison, the results obtained from µCT systems were used as comparative values to validate the usage of CBCT imaging techniques.

## 2. Materials and Methods

### 2.1. Teeth Selection Criteria and Preparation

In the present study, extracted human mandibular first and second molars (n = 50) were included. The teeth were collected from the Clinic of Oral and Maxillofacial surgery as well as from private dental practices. They were extracted for medically justifiable reasons without any connection to the present study. No information was available about the patients’ name, age, sex and general health condition. Exclusion criteria were the following: endodontically treated teeth, teeth with incomplete root development, root resorptions, fractured roots and teeth carious lesions. All molars were stored in 0.1% thymol solution at 4 °C. The teeth were cleaned from calculus as well as from hard and soft tissue. The tomographic imaging was performed only with the already extracted teeth ex vivo. The selected distal root canals were evaluated by visual inspection of their volume models and showed no signs of bifurcations and ramifications.

### 2.2. Imaging Procedures and Evaluation of Centre Line

All teeth were scanned by means of CBCT and µCT systems. The three-dimensional radiographs were performed by means of CBCT unit (3D Accuitomo, Model: MCT-1 Type: EX-2F8, Morita Europe GmbH, Dietzenbach, Germany) and µCT scanner (in situ computed tomography scanner FCTS 160-IS; Finetec GmbH, Garbsen, Germany) (Table 1). During the image recording, eight teeth were fixed in a cube made of extruded polystyrene rigid foam and were simultaneously scanned. For all devices, a correction procedure was carried out to correct variations in the pixel sensitivity of the cameras before the scanning process.

**Figure 1:**
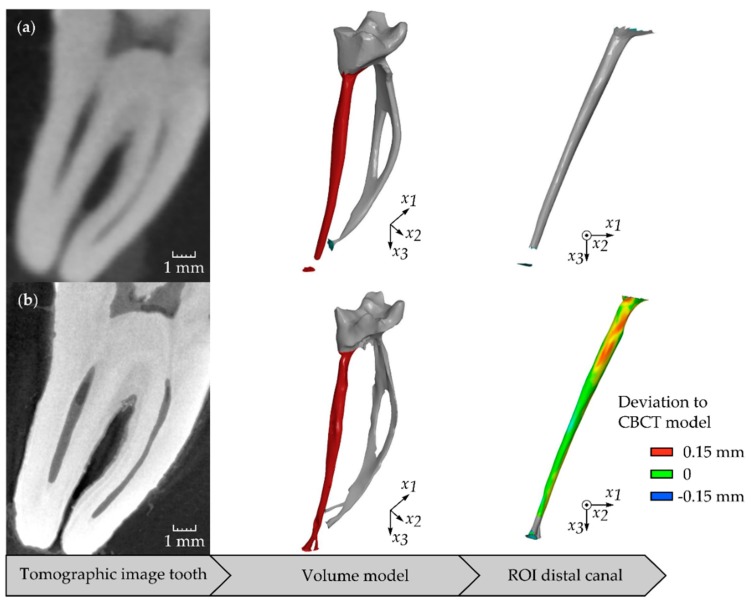
Buccolingual view of a mandibular first molar taken by topographical imaging techniques, the resulting volume model of root canal morphology from three-dimensional imaging data and its distal root canal: (**a**) CBCT, (**b**) µCT.

For CBCT and µCT, all images were imported in a digital imaging and communications in medicine (DICOM) file format and evaluated in analysing and visualisation software for industrial computed tomography data (VGStudio Max 2.2.6, Volume Graphics, Heidelberg, Germany). A suitable greyscale threshold, guaranteeing the determination of the correct distal canal’s boundary as well as a low image noise, was chosen for the image evaluation. The resulting binary images were used to reconstruct three-dimensional volume models (Figure 1).

**Figure 2:**
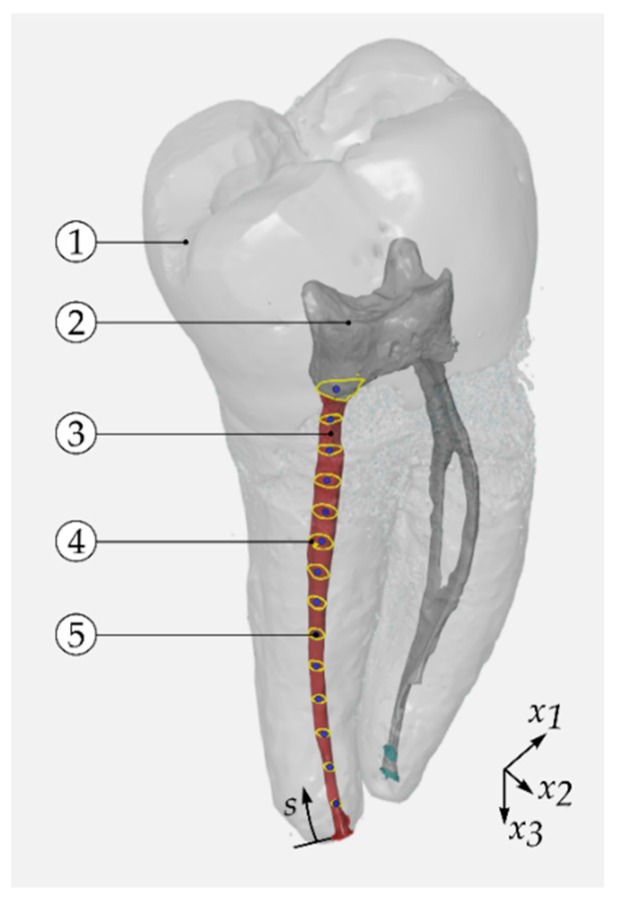
Exemplarily determination of centroids (reduced number slices per long axis of tooth): outer contour of first molar, **1**; root canal morphology, **2**; distal root canal (region of interest (ROI), red surface), **3**; root canal’s cross-section slice (yellow lines), **4**; centroid (blue points), **5**.

As stereolithography (STL) files, the data of the volume models were exported from VGStudio and post-processed by means of an analysis software for three-dimensional optical measurement (GOM Inspect, Braunschweig, Germany). The volume models were cut along the longitudinal axis of the root canal every 10 µm to obtain parallel sections, called slices (compare Figure 2 with reduced number of slices). The distal root represented the region of interest (ROI). Using a multi-paradigm numerical computing environment (MATLAB 9.5, MathWorks, Inc., Natick, MA, USA), the appropriate cross sections in each slice, representing the outer boundary of the root canal without artefacts, were used to determine the centroids at its position in longitudinal root canal axis $x_{3}$ (compare Figure 2). Incomplete cross-sections as well as the artefacts were removed manually. The connection of the centroids of each slice j were considered as the centre line:

(1) $$x_{i}^{j}\;=\;{\left(x_{1}^{j},x_{2}^{j},x_{3}^{j}\right)}^{T}\;=\;{\left(x_{1}\left(s^{j}\right),x_{2}\left(s^{j}\right),x_{3}\left(s^{j}\right)\right)}^{T}\in {\mathbb{R}}^{3},$$

where the arc length s was oriented from the apical foramen to the floor of the pulp chamber. The apical foramen was chosen as the starting point because it was an explicitly defined reference point at the end of the canal. This yielded $x_{i}\left(s^{j}\right),$ in the following denoted as $x_{i}\left(s\right)$. The coordinate $x_{1}$ represented the width in buccolingual direction, $x_{2}$ was the width in mesiodistal direction and $x_{3}$ was the direction in the tooth’ longitudinal axis.

### 2.3. Filtering and Measurement Algorithm of Centre Line

**Figure 3:**
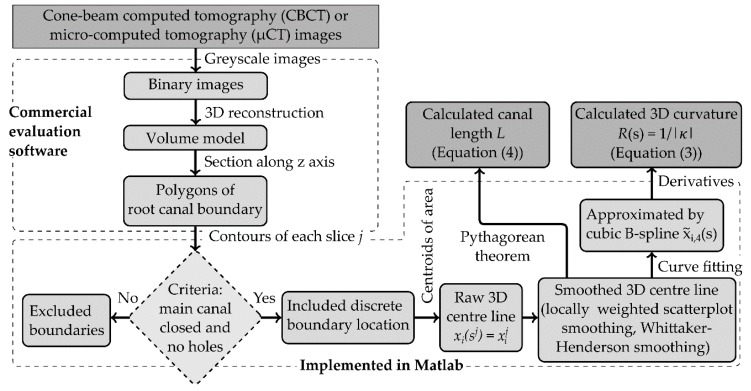
Schematic of used commercial software and implemented evaluation algorithm.

Using the closed root canal sections of each slice, the raw centre lines were determined (Figure 2 and Figure 3). The centroids were linear interpolated to equidistant increments of line segment with a length of Δs = 10 µm. To remove high-frequency fluctuations, the coordinates $x_{1}\left(s\right)$ and $x_{2}\left(s\right)$ were filtered by means of locally weighted scatterplot smoothing (LOWESS) [23]. For this filter, a span w was defined.

All components $x_{i}\left(s\right)$ were approximated by cubic B-spline curves:

(2) $${\tilde{x}}_{i,p}\left(s\right)=\overset{N_{k}}{\underset{k=1}{\sum }}\alpha_{k}B_{k,p}\left(s\right),$$

where $\alpha_{k}$ were the B-spline coefficients, $B_{k,p}\left(t\right)$ was the kth B-spline, $N_{k}$ were the number of data points for the calculation by means of least squares approximation and p was the polynomial degree, which was set to p = 4 (compare Reference [9]). Using the approximation ${\tilde{x}}_{i}\left(s\right)\;=\;{\tilde{x}}_{i,4}\left(s\right)$, the radius of curvature was determined. The radius of curvature was denoted as:

(3) $$R\left(s\right)=1/\kappa (s)\;={\left|{\dot{\tilde{x}}}_{i}\left(s\right)\right|}^{3}/\left|\dot{{\tilde{x}}_{i}}\left(s\right)\times {\ddot{\tilde{x}}}_{i}\left(s\right)\right|,$$

where ${\dot{\tilde{x}}}_{i}=\partial \left({\tilde{x}}_{i}\right)/\partial s$ and κ(s) was the root canal curvature. The deviations were determined directly by means of the B-spline curve, which were smoothed by means of the Whittaker–Henderson smoothing algorithm with a basic smoothing parameter σ∈(0, 1) [24]. The total arc length of the root canal was calculated using the Pythagorean theorem of the filtered centroids defined as:

(4) $$L=\overset{N_{j}}{\underset{j=1}{\sum }}\sqrt{\overset{3}{\underset{i=1}{\sum }}{\left(x_{i}^{j+1}-x_{i}^{j}\right)}^{2}},$$

where $x_{i}\left(s^{j}\right)=x_{i}^{j}$ and $x_{i}\left(s^{j+1}\right)=x_{i}^{j+1}$ were two consecutive points at the positions $s^{j}$ and $s^{j+1}\;$ and $N_{j}$ was the number of slices of each root canal.

### 2.4. Data Analysis

The canal curvature was evaluated as a function of canal length. To compare the distal root canals with different total lengths L, the canal length s was normalised by L. The normalised length was divided into three equally spaced parts. In the following, the parts were denoted as apical, medial and coronal third.

The quartile deviation $\delta \left(\cdot \right)=0.5\left(q_{75}\left(\cdot \right)-q_{25}\left(\cdot \right)\right)$ of canal curvature and radius of curvature were determined using the calculated canal curvature and radius of curvature, respectively. The values $q_{25}$ and $q_{75}$ were the first and third quantile.

### 2.5. Algorithm Performance

**Figure 4:**
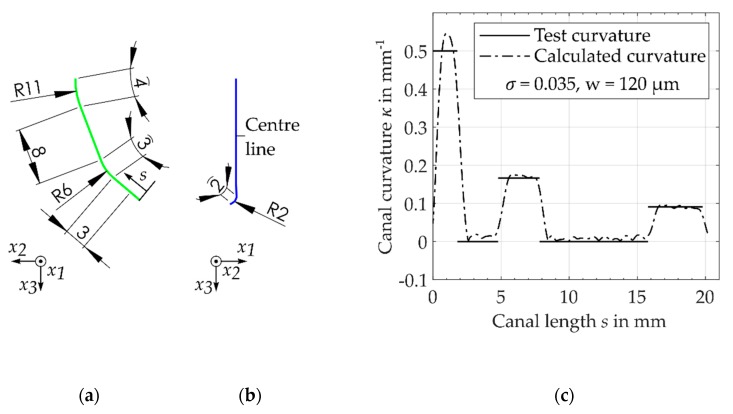
Centre line of a defined artificial root canal: (**a**) buccolingual view, (**b**) mesiodistal view, (**c**) result of developed algorithm and the defined canal curvature of the defined test case.

For the verification of the measurement algorithm, an artificial root canal with a known curvature $\kappa_{REF}$ was defined as the test case. Considering the distribution of the radius of curvature as measured by Kirsch et al. [3], the total arc length of the centre line was 20 mm. In the buccolingual view, the line was curved with a radius of 11 mm (high radius of curvature) and 6 mm (most common radius of curvature). In the mesiodistal view, the centre’s line ending had a small radius of 2 mm (Figure 4b). To get a more realistic test case, a normally distributed random error with an amplitude of $\Delta x_{1}=\Delta x_{2}=10$ µm was introduced for the directions $x_{1}$ and $x_{2}$. As described above, for an accurate estimation of the canal curvature and canal length, a set of feasible parameters w and σ was required.

**Figure 5:**
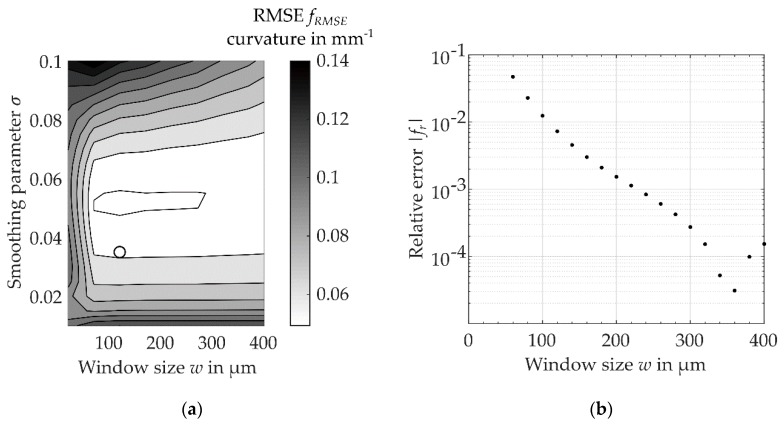
Error of canal curvature and length using the implemented evaluation algorithm for the centre line of the artificial root canal with random error in both transversal directions $x_{1}$ and $x_{2}$: (**a**) root mean square error (RMSE) for different smoothing parameters σ and w with selected parameters (circle, σ = 0.035, w = 120 µm), (**b**) relative error $f_{r}$ of the calculated canal length for different window sizes w.

Due to fluctuation in the cross-sectional coordinates $x_{1}$ and $x_{2}$, the calculated arc length s depended on the given signal-to-noise ratio, which was caused by a slightly incorrect estimation of centroids. The usage of filters reduced the relative error $f_{r}=\left(L-L_{ref}\right)/L_{ref}$ of the determined canal length. For a window size of w = 120 µm, the resulting error was $f_{r}$ = 0.7% (Figure 5a). Starting from w = 370 µm, the error increased due to the increasing shortening of the total canal length and the associated negative relative error. For the given range of smoothing parameter σ = [0.01, 0.1] and a window size of w = [10 µm, 400 µm], the root mean square error (RMSE) $f_{RSME}$ of the curvature was calculated (Figure 5b). For σ = 0.035 and w = 120 µm, a relatively smooth measurement of curvature with a small error of $f_{RMSE}$ = 0.058 mm^−1^ resulted (Figure 4c and Figure 5a). At the same time, the used value w enabled an accurate calculation of the canal length. Using these parameters, a suitable measurement of the canal curvature as well as the canal length of the defined test case was obtained. Thus, in the following, these parameters were used to quantify the geometry of the root canal’s centre line.

## 3. Results

### 3.1. Comparison of Three-Dimensional Centre Lines

**Figure 6:**
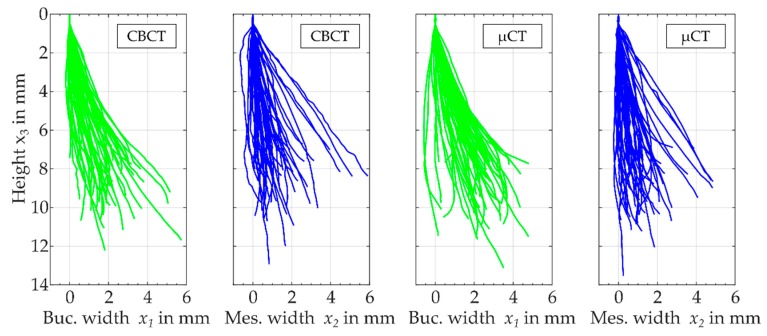
Buccolingual view and mesiodistal view of the filtered centre lines $x_{i}\left(s\right)$ evaluated from CBCT images and µCT images for all investigated root canals.

For all resulting root canal’s centre lines, a segment with a length of 0.5 mm was aligned parallel to the $x_{3}$-axis in the buccolingual and mesiodistal view (Figure 6). It can be seen that the main dimensions of both imaging techniques resulted in values up to 14 mm in height direction and 6 mm in width direction. In general, all centre lines had comparable dimensions in the buccolingual direction as well in the mesiodistal direction. Furthermore, the three-dimensionality could be observed since a significant width in both transversal directions $x_{1}$ and $x_{2}$ existed. Depending on the imaging technique used, the concrete course of the centre line varied slightly.

### 3.2. Radiological Three-Dimensional Root Canal Length

The length of the investigated root canals differed between 7.1 and 13.4 mm (CBCT) and between 7.2 mm and 13.9 mm (µCT). For CBCT, the median of the canal lengths was 9.6 mm (δ(L) = 0.9 mm) and for µCT, 10.2 mm (δ(L) = 0.9 mm), respectively. The distribution of canal length depended on the imaging technique used. Considering the results gained by means of CBCT, the highest probability was obtained by root canals with a length between 10 mm and 10.5 mm with a relative probability of p = 16%. For the imaging technique µCT, the high probability was p = 20% for a length between 9.5 mm and 10 mm. Considering the cumulative distribution function (CDF), the length from CBCT images and the lengths measured using µCT images were normally distributed. In general, as expected, the total lengths gained from µCT images were slightly longer than the lengths determined by CBCT.

### 3.3. Curvature Measurements

**Figure 7:**
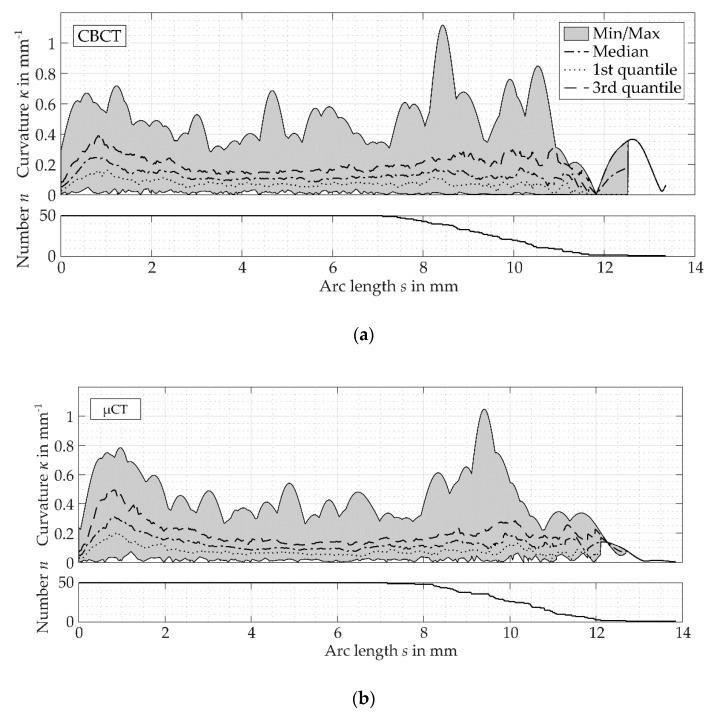
Measurements of root canal curvature κ(s) and the number n(s) of root canals with a maximum length up to this point s calculated by means of: (**a**) CBCT images, (**b**) µCT images.

Considering the median of the curvature’s path calculated from CBCT and µCT images, stronger curvatures can be seen close to the apical foramen (Figure 7). This segment was followed by a section of weak and uniform curvatures. Starting at approximately s = 7 mm, the canal curvatures increased and showed a more alternating course. The quartile deviation increased in the segment 0≤s≤2 mm and in the segment 7 mm ≤s≤L. The reduction of the enclosed areas between the minimum and the maximum curvature at the canal’s ends could be argued by the decrease of the evaluable canals’ number with a total length up to this value.

The median value of the root canal curvatures obtained similar results for the evaluation of CBCT and µCT images (Table 2). For both imaging techniques, the apical third had the highest curvatures, and the smallest curvatures were measured in the medial third. Furthermore, the median values showed the highest deviation of curvatures in the apical third (Table 2).

The values of maximum curvatures evaluated from CBCT images were 17% smaller than the results obtained by means of µCT images (Table 3). The maximum curvatures derived from CBCT and µCT images were located with the highest probability in the apical third and with the second highest probability in the coronal third. The medial part represented the section with the smallest curvatures.

The evaluation of the maximum curvature for each third showed that the median value evaluated from CBCT images is 13% smaller in the apical third, 16% higher in the medial third and 15% higher in the coronal third (Table 4). Evaluating the images from the CBCT system, the median value of the maximum curvatures obtained higher values in the coronal and medial third, whereas the median in the apical third showed the minimum radius of curvature determined by means of the µCT system.

## 4. Discussion

The three-dimensional radiologic imaging techniques, CBCT and µCT, were used to determine the root canal curvature of the distal root of human mandibular first and second molars. Considering the distal main root canal, the centre line was calculated using the centroids of root canal’s slices of the segmented volume models. Due to the selection of canals without the tomographically visible presence of a bifurcation and/or ramifications, this approach was appropriate to calculate the centre line. Another method for the determination of the centre line is the morphological thinning of the root’s volume model, which offers the possibility to obtain quasi-centrally located centre points of the main canal as well as of the bifurcation and ramifications, but entails more computational costs [13]. Since iterative thinning has an effect on all directions of the root canal, the lengths of all ends are shorter due to the iterative reduction of the outer voxels. Furthermore, this approach can result in several short branches connected to high curvature points of the reconstructed three-dimensional object’s surface [13]. Thus, several investigations described the centre line as a connection of centroids’ perpendicularly cut root canal slices [8,12,14,15].

The curvature measurement in the present study resulted in similar curvatures compared to already existing studies, that determined the radii by a circular approximation of the root canal [3] or evaluated the rate of turning of the tangent vector at a given point [9]. The length was normalised by the total length of the root canal and was divided into three parts. Lee et al. [9] and Choi et al. [17] used the same sub-divisional approach and segmented the canal length into cervical, middle and apical thirds to compare the results from a number of 46 intact maxillary molars and 108 mandibular molar’s distolingual roots, respectively. Compared to this study, Lee et al. measured a similar relative probability of the highest curvatures, whereas Choi et al. obtained different results. Choi et al. defined the curvature by the slope of a line connecting two adjacent points [17]. The standardisation using the total canal length, as in the present study, enabled a statistical comparison of canals with different lengths. However, a description of the curvature as a function of the arc length could have more clinical relevance for practical use. Due to the knowledge of the maximum root canal curvature and its location, critical regions of the root canal could be identified, and thus, the success of endodontic treatments could be improved. For this reason, the locations of the maximum curvatures were additionally determined in this study.

Considering the total length of the root canal, Kirsch et al. obtained different values for 100 extracted human mandibular first and second molars due to the definition of the arc length from the apical foramen to the orifice as well as to the top of the access cavity [3]. Lee et al. analysed 37 first molar and determined an averaged length from the apex to the orifice of 10.92 mm for mesiobuccal root canal and 10.79 mm for mesiolingual root canals [10], which were comparable to the results of the present study.

Effects such as image noise, interpolation errors of volume model or false detected canal boundaries caused fluctuations in both transversal directions. Peters et al. obtained similar effects for the measurement of the internal sphere’s maximum diameter [8,25]. The reduction of data points could help to avoid this issue (compare References [9,17]) but at the same time, it also reduces the minimal measurable radii of curvature. Using a smoothing algorithm with a fixed window size for both transversal coordinates of the centre line and a Whittaker–Henderson smoothing algorithm, the fluctuations were reduced. A test case of a three-dimensional curved root canal was designed to obtain suitable smoothing parameters. The test case contains common curvatures, as well as low and high curvatures, as measured by Kirsch et al. [3]. The implementation of a filter algorithm and the choice of appropriate smoothing parameters obtained a good agreement of curvature measurement to the curvatures of the test case. However, an error was measured at discontinuous sections of curvature graphs. For steadier centre lines of naturally curved root canal, a better approximation technique is desired.

### Strengths and Limitations

The measurement of the three-dimensional continuous root canal curvature enabled the identification of critically curved canal sections and a reliable quantification of its radii of curvature (compare References [3,9,17]). Compared to the evaluation of two-dimensional radiographic images, the consideration of the three-dimensional curvature obtained more realistic results due to the three-dimensional curved human root canals. In contrast, two-dimensional stepwise methods of curvature measurement, such as the approximation by lines converging to the root canals centre line and the determination of curvatures by means of angles or a circular approximation, were not necessary.

Considering the statistical evaluation, the median values of curvature were similar to the evaluation of CBCT and µCT images. The maximum curvature and its location differed for both techniques due to different resolutions of the tomographic images. Nevertheless, the radiation dose of the three-dimensional tomographic imaging technique µCT is generally higher than the dose of CBCT and thus, µCT systems are used only ex vivo/in vitro for endodontic research at the moment. Using CBCT systems, curvature measurements could also be performed in vivo (compare Reference [22]), and the access to larger amounts of clinical databases for clinical studies could be provided due to its clinical relevance.

Taking the quantification of curvature and the radiation dose into account, the usage of CBCT systems in connection with the demonstrated approach of a three-dimensional curvature measurement seems to be a promising approach for practical usage in endodontic treatments, especially for difficult cases. The advantages of the possibility to illustrate the three-dimensionality of the root canal centre lines in the buccolingual and mesiodistal direction as well as the identification of critically curved sections definitely outweigh the drawbacks of sufficiently accurate curvature measurements in critical endodontic treatment cases. The present study demonstrated the clinical relevance and the implementation of the continuous three-dimensional measurement of root canal curvature by means of CBCT systems.

## 5. Conclusions

The validation of an approach of the continuous three-dimensional curvature measurement determined from three-dimensional imaging data was successfully demonstrated. Both investigated tomographic techniques, CBCT and µCT, were suitable for providing the necessary tomographic images and measuring the canal curvature. Since the evaluation of CBCT images obtained statistically similar results to the evaluation of the high-resolution µCT images, the application of CBCT imaging techniques for the measurement of canal curvature seems to be a sufficient and promising approach for a reliable measurement of three-dimensionally curved human root canals. The lower radiation exposure of CBCT systems and the possibility of the imaging in vivo were the main advantages and highlighted the clinical relevance of the determination of root canal curvature in critical endodontic treatment cases by currently available CBCT images without the necessity of a higher-resolution imaging device.

## Figures and Tables

**Table 1:** Parameters of applied radiologic imaging techniques.

Parameters	Unit	CBCT	µCT
Resolution	mm/pixel	0.08	0.021
Tube voltage	kV	70	80
Tube current	mA	3	0.08
Exposure time	s	17.5	900
Source object distance	mm	500	150

Cone-beam computed tomography (CBCT); micro-computed tomography (µCT).

**Table 2:** Median and quartile deviation of canal curvatures (n = 50).

Imaging Technique	Apical Third	Medial Third	Coronal Third	Overall
CBCT	0.15 {0.05} (6.7 {2.2})	0.1 {0.03} (10 {2.3})	0.12 {0.02} (8.5 {1.7})	0.12 {0.02} (8.1 {1.5})
µCT	0.16 {0.06} (6.3 {2.3})	0.09 {0.02} (10.6 {2.6})	0.12 {0.02} (8.4 {1.8})	0.12 {0.02} (8.5 {1.8})

Canal curvature κ in mm^−^^1^; ( ) radius R of curvature in mm; { } quartile deviation.

**Table 3:** Median and quartile deviation of the maximum curvatures and its relative probability in the different thirds (n = 50).

Imaging Technique	Maximum Curvature	Apical Third	Medial Third	Coronal Third	Location s in mm
CBCT	0.38 {0.13} (2.6 {0.7})	56%	6%	38%	2.5 {3.3}
µCT	0.47 {0.16} (2.1 {0.8})	68%	8%	24%	1.4 {2.5}

Canal curvature κ in mm^−^^1^; ( ) radius R of curvature in mm; { } quartile deviation.

**Table 4:** Median and quartile deviation of the maximum curvatures.

Imaging Technique	Maximum Curvature	Location s in mm
Apical third		
CBCT	0.33 {0.11} (3 {1})	1.2 {0.7}
µCT	0.38 {0.13} (2.6 {0.8})	0.9 {0.4}
Medial third		
CBCT	0.21 {0.05} (4.7 {1.2})	5 {1.1}
µCT	0.18 {0.04} (5.4 {1.1})	5 {1.2}
Coronal third		
CBCT	0.31 {0.08} (3.2 {1.3})	8.2 {1.1}
µCT	0.27 {0.08} (3.7 {1.3})	9.1 {1}

Canal curvature κ in mm^−^^1^; ( ) radius R of curvature in mm; { } quartile deviation.
